# Traditional Growth-Friendly Implants Result in Improved Health-Related Quality of Life in Cerebral Palsy Patients with Early-Onset Scoliosis

**DOI:** 10.3390/jpm15110506

**Published:** 2025-10-24

**Authors:** Nicholas J. Buckler, Margaret Sun, Mason Al Nouri, Jason J. Howard, Majella Vaughan, Tricia St. Hilaire, Hiroko Matsumoto, Paul D. Sponseller, John T. Smith, George H. Thompson, Pediatric Spine Study Group, Ron El-Hawary

**Affiliations:** 1 Faculty of Medicine, Dalhousie University, Halifax, NS B3K 6R8, Canada; nicholas.buckler@dal.ca (N.J.B.); msun33@uwo.ca (M.S.); 2 IWK Health Centre, Halifax, NS B3K 6R8, Canada; mya2002@qatar-med.cornell.edu; 3 Nemours/Alfred I. duPont Hospital for Children, Wilmington, DE 19803, USA; jason.howard@iwk.nshealth.ca; 4 Pediatric Spine Foundation, Valley Forge, PA 19481, USA; mvaughan@pediatricspine.org (M.V.); tsthilaire@pediatricspine.org (T.S.H.); rwoon@pediatricspine.org; 5 Boston Children’s Hospital, Boston, MA 02115, USA; hiroko.matsumoto@childrens.harvard.edu; 6 Department of Orthopedic Surgery, Johns Hopkins University, Baltimore, MD 21287, USA; psponse@jhmi.edu; 7 Primary Children’s Hospital, Salt Lake City, UT 84113, USA; john.smith@hsc.utah.edu; 8 Rainbow Babies & Children’s Hospital, Cleveland, OH 44106, USA; george.thompson@uhhospitals.org

**Keywords:** cerebral palsy, early onset scoliosis, growth friendly, health-related quality of life, MCGR, MAGEC, VEPTR, personalized medicine

## Abstract

**Background/Objectives**: In an effort to promote personalized medicine, the purpose was to (1) analyze health-related quality of life (HRQoL) in cerebral palsy (CP) patients treated with growth-friendly implants for early-onset scoliosis (EOS), and (2) compare traditional implants (traditional growing rods [TGRs], VEPTR) with magnetically controlled growing rods (MCGRs). **Methods**: Twenty-four patients with CP and EOS were identified from an international multicenter database. Mean EOSQ-24 domain and total scores and absolute differences from pre-index surgery to the minimum two-year follow-up were compared. **Results**: For all patients: Pre-index surgery EOSQ-24 total score: 48.9 vs. follow-up: 53.8. Follow-up scores were greater than at pre-op for 10 of the 12 domains, with the only significant difference being activities of daily living. Growth-friendly implants had positive absolute differences for 8 of the 12 domains and in the total score. Nine traditional implant patients had a pre-index surgery EOSQ-24 total of 45.8 points, while 15 MCGRs patients had a score of 50.8 points. At follow-up, traditional implant patients had greater scores than at pre-index surgery for all 12 domains, with total score of 55.1 points, and positive absolute differences for all domains (non-significant). MCGRs had greater scores than at pre-index surgery for six domains, with a total score of 53.1 points (non-significant), and positive absolute differences for seven domains. Traditional implants had a significantly greater absolute difference for emotion than MCGRs (*p* = 0.030). **Conclusions**: At the minimum two-year follow-up, CP patients had small, but statistically non-significant, improvements in HRQoL following growth-friendly surgery. Compared to MCGRs, traditional implants provided a modest additional benefit in HRQoL.

## 1. Introduction

Cerebral palsy (CP) is defined as a group of permanent disorders of the development of movement and posture that are caused by interruptions during fetal or infant brain development [1,2]. This results in motor disorders, activity limitations, and secondary musculoskeletal problems. Scoliosis is defined as lateral curvature of the spine greater than 10°, with the term “early-onset scoliosis” (EOS) reserved for patients with scoliosis that is present before the age of 10 years (9 years of age or younger), regardless of etiology [3]. Although the onset of scoliosis in CP is typically later than 8 years, a smaller subset of younger patients of advanced disease severity can develop clinically significant scoliosis that may lead to seating imbalance, pain, decubitus ulcers, and pulmonary dysfunction [4]. These curves are usually progressive and tend to become more rigid with time [5]. Approximately 50% to 75% of pediatric patients with advanced levels (Gross Motor Function Classification System IV–V) of CP are affected by spinal deformity, with scoliosis occurring in between 21% and 64% [6,7,8].

There is an increasing interest in treating CP patients with EOS utilizing growth-friendly implants, though there have been few studies which have specifically targeted this population. The goal of these implants in CP is to prevent curve progression, improve seating tolerance, decrease pain, and promote lung development, factors associated with improved health-related quality of life (HRQoL) in older children who have undergone definitive fusion [. It is theorized that similar improvements in HRQoL will be seen after growth-friendly implants in CP [9].

Minimal HRQoL research has been conducted on EOS [10], although it is arguably the most important clinical outcome, particularly for children with CP and scoliosis. HRQoL is a challenging outcome to analyze, as the subjective measurements can vary with time and between patients and their parents/caregivers based on individual priorities [7,8]. An analysis of HRQoL can be a beneficial aspect of patient management, along with the more objective radiographic parameters that are commonly evaluated for EOS in patients with CP. A tool has been specifically developed for measuring HRQoL in EOS patients. The 24-item Early-Onset Scoliosis Questionnaire (EOSQ-24) includes 24 items or questions that are completed by patient caregivers [11]. This validated questionnaire evaluates a number of aspects of HRQoL, such as pain, pulmonary and physical function, daily living, and emotion, as well as family burden and satisfaction.

**Figure 1:**
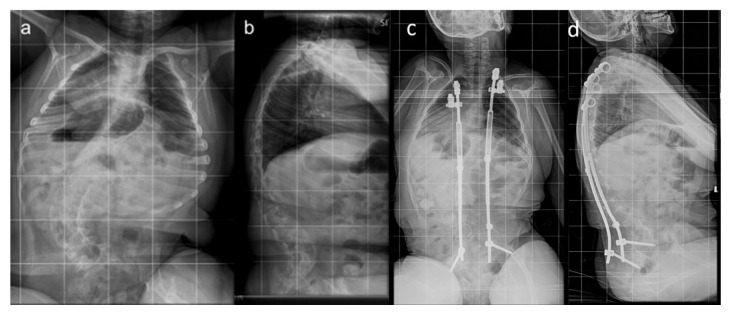
Eight-year-old male VEPTR^TM^ patient EOS secondary to GMFCS V cerebral palsy. Patient had undergone five lengthening procedures by four years post-implantation. He did not experience any post-operative complications and his five-year follow-up EOSQ-24 total score was 61/100. (**a**) Pre-operative posteroanterior radiograph demonstrating 72-degree scoliosis. (**b**) Pre-operative lateral radiograph. (**c**) Four-year post-implantation anteroposterior radiograph. (**d**) Four-year post-implantation lateral radiograph.

**Figure 2:**
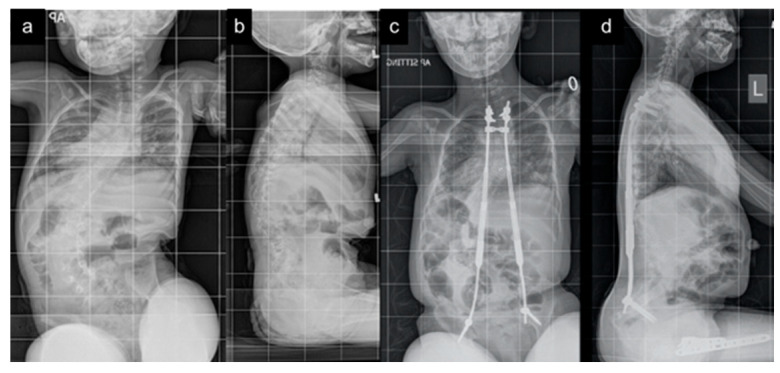
Five-year-old female MCGRs patient with EOS secondary to GMFCS V cerebral palsy. Patient underwent only one surgery for implantation and had magnetic lengthenings in clinic every three months. No complications. Pre-operative EOSQ-24 total score of 45/100 and four-year follow-up score of 57/100. (**a**) Pre-operative anteroposterior radiograph demonstrating 76-degree scoliosis. (**b**) Pre-operative lateral radiograph. (**c**) Four-year post-implantation anteroposterior radiograph. (**d**) Four-year post-implantation lateral radiograph.

Growth-friendly implants include the use of traditional growing rods (TGRs), the vertically expandable prosthetic titanium rib (VEPTR^TM^, DePuy Synthes Spine, Inc., Raynham, MA, USA) (Figure 1), as well as magnetically controlled growing rods (MCGRs, MAGEC, NuVasive Spine, San Diego, CA, USA) (Figure 2). MCGRs allow for periodic non-invasive lengthening without the need for the open surgery approach required for traditional implants like TGRs and the VEPTR [12]. This technology also helps protect children from the negative effects of repetitive general anesthesia and surgical interventions.

HRQoL outcomes in CP patients treated with growth-friendly implants for EOS have not yet been described. The purpose of this study was to promote personalized medicine by analyzing these outcomes in cerebral palsy patients treated with growth-friendly implants for EOS. We hypothesize that in CP patients treated with growth-friendly implants for EOS, HRQoL will improve at the minimum two-year post-index surgery follow-up, and MCGRs will provide better HRQoL outcomes than traditional implants.

## 2. Materials and Methods

This was a retrospective review of prospectively collected data on patients from the Pediatric Spine Study Group (PSSG), an international, multicenter EOS database. Patients less than 10 years old (9 years of age or younger) with a diagnosis of CP and EOS were included if they had no previous spine surgery, were treated with growth-friendly implants (either traditional implants or MCGRs), had potential for a minimum two-year follow-up, and did not have converted implant systems. We then excluded those that did not have EOSQ-24 questionnaires, completed both pre-index surgery and at the follow-up, a minimum of two years post-index surgery. Data collection timeframe was patients who had index surgery between 18 April 2012–17 February 2017.

The EOSQ-24, specifically developed for measuring HRQoL in EOS patients, includes 24 items or questions that are completed by patient caregivers [9]. We used this validated questionnaire as the primary outcome to evaluate multiple aspects of HRQoL in our study. Secondary outcome measures included radiographic parameters (primary curve angle, maximum/global kyphosis, T1-S1 height, and T1-T12 height), gender, prior surgical or brace treatment, GMFCS level, age at index surgery, and follow-up period.

For EOSQ-24 data, raw scores for each item were transformed to domain scale scores that range from 0 (poor) to 100 (excellent). Means were calculated for each domain and the means of all 12 domains (total score) at pre-index surgery and at the minimum two-year post-index surgery follow-up for all patients were included, as well as those for both treatment groups (traditional implants and MCGRs). These values were then compared between time points and growth-friendly implant type. Mean absolute differences from pre-index surgery to the minimum two-year follow-up were calculated for each domain for every individual patient, averaged by domain, and then compared between treatment groups. Distribution analysis was performed, and all assumptions were met. For secondary outcome measures, descriptive statistics were utilized. All mean values were reported with 95% confidence intervals.

Comparisons between means were conducted using independent samples two-tailed *t* tests with equal variances not assumed, using SPSS software (Version 25–IBM; Armonk, NY, USA). Statistical significance was accepted at *p* < 0.05. To strengthen the results of our study, we followed the items from the STROBE checklist.

## 3. Results

A total of 24 patients (13 females, 11 males) met the inclusion criteria and had both pre-index surgery and the minimum two-year post-index surgery follow-up EOSQ-24. Among these, 5 patients had previously undergone bracing treatment, 21 (84%) were GMFCS V, the mean age at index surgery was 7.4 years (CI: 6.6–8.2 years), and the mean follow-up was 3.5 years (CI: 3.0–3.9 years) (Table 1).

**Figure 3:**
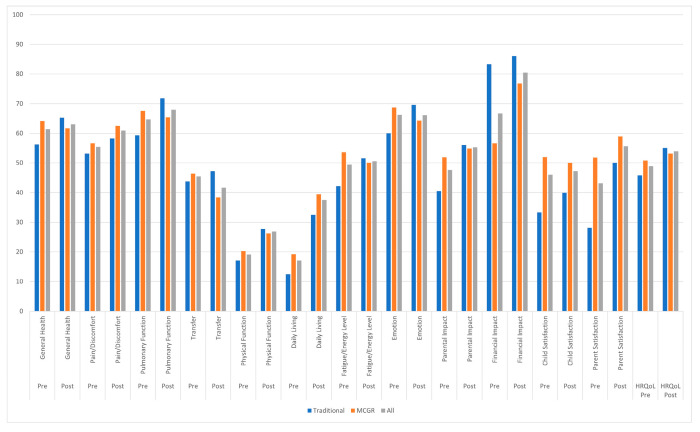
Mean EOSQ-24 scores for all domains at pre-index surgery and at minimum two-year post-index surgery follow-up for patients treated with traditional implants (n = 9), magnetically controlled growing rods (MCGRs) (n = 15), and all patients treated with growth-friendly implants (n = 24).

Nine patients (38%) were treated with traditional implants. Among these, five patients were treated with VEPTR and four with TGRs, all were GMFCS V, the mean age at index surgery was 7.1 years (CI: 5.8–8.4 years), and the mean follow-up was 4.4 years (CI: 3.7–5.2 years). Analyses between VEPTR and TGRs patients were conducted for the pre-index surgery scores, the minimum two-year post-index surgery follow-up scores, and the absolute differences, with the only statistically significant difference for the minimum two-year post-index surgery follow-up being financial impact, which was less of a burden in VEPTR patients (*p* = 0.015). Fifteen patients (62%) were treated with MCGRs. Among these, 12 patients (80%) were GMFCS V, the mean age at index surgery was 7.7 years (CI: 6.6–8.7 years), and the mean follow-up was 2.9 years (CI: 2.6–3.2 years). MCGRs patients had a statistically significantly shorter follow-up time than patients treated with traditional implants (*p* = 0.003). All growth-friendly implant patients had a pre-index surgery mean primary curve of 81.2° (CI: 73.1°–89.4°) (Table 2) and a mean EOSQ-24 total score of 48.9 points (CI: 40.6–57.3 points). The minimum two-year post-index surgery follow-up scores were greater than at pre-index surgery for 10 of the 12 domains, with a mean total score of 53.8 points (CI: 46.1–61.6 points), but only the activities of daily living domain achieved a statistically significant difference (*p* = 0.048) (Figure 3).

At pre-index surgery, traditional implant patients had a mean primary curve of 71.8° (CI: 59.4°–84.1°) (Table 2) and a mean EOSQ-24 total score of 45.8 points (CI: 32.4–59.2 points) (Figure 3). The minimum two-year post-index surgery follow-up scores were greater than the pre-operative scores for all 12 domains, with a mean total score of 55.1 points (CI: 45.7–64.6 points) (all non-significant). When traditional implant patients were divided up into VEPTR and TGRs, there were no significant differences between these implant types at pre-index surgery. The only significant difference at follow-up was in the financial impact domain, which was more burdensome in TGRs patients. Changes in individual domains were variable between these two implant types; however, both groups had greater total scores at follow-up than at pre-index surgery.

We compared the mean score for each domain at pre-index surgery between traditional implants and MCGRs patients, with the only significant difference being less burdensome financial impact with traditional implants (*p* = 0.046). No statistically significant differences were found for any domain between growth-friendly implant types at the minimum two-year follow-up.

All growth-friendly implant patients had positive mean absolute differences between the minimum two-year follow-up and pre-index surgery for 8 of the 12 EOSQ-24 domains and for the total score (Table 3). Traditional implant patients had a positive mean absolute difference for all 12 domains and the total score. MCGRs patients had positive values for 7 of the 12 domains and for the total score. Traditional implants had a significantly greater absolute difference for emotion when compared with MCGRs (*p* = 0.030). Mean absolute differences for all 11 other domains were not significantly different between treatment groups.

## 4. Discussion

Growing rods can be an effective method of treating scoliosis in children with CP, allowing for a delay in definitive fusion until skeletal maturity to allow for spinal and thoracic growth. Given the increasing utilization of growth-friendly implants in children with CP and EOS, it is important to understand whether the initiation of this “surgical journey”—whereby multiple operations either for device lengthenings and/or revisions will be invariably required—for these patients who are often medically fragile is warranted, in view of the potential risks involved. One of the potential benefits of growth-friendly implants in CP patients involves the potential for HRQoL improvement, arguably the most important factor for these children. In older children with CP, scoliosis surgery has been shown to improve HRQoL, as measured by the CPCHILD questionnaire [5,13]. We hypothesized that HRQoL would be improved at the minimum two-year post-index surgery follow-up compared to pre-index surgery, and that MCGRs would provide greater HRQoL than traditional implants. In the current study, however, we found that CP patients had only small, statistically non-significant improvements in HRQoL following growth-friendly implants for EOS. In addition, despite the theoretical benefit of reducing the need for device lengthenings under general anesthesia, aside from the ‘emotion’ domain, MCGRs were not found to be associated with improved HRQoL when compared to traditional implants.

Bauer et al. compared pre-operative and post-operative EOSQ-24 scores in 302 EOS patients of various etiologies and also found no significant difference between TGRs and MCGRs groups for most domains [14]. In their study, MCGRs showed significant improvement versus TGRs in only the ‘transfer and fatigue/energy’ level domains. In another study comparing treatment with MCGRs versus TGRs in patients of any etiology, MCGRs showed significantly greater EOSQ-24 scores for financial burden and overall satisfaction domains [10]. However, once these scores were controlled for the duration of follow-up, there were no longer any significant differences between treatment types. Likewise, MCGRs patients in our study had a significantly shorter mean follow-up time than traditional implant systems (*p* = 0.003) (Table 1), which may have impacted their ability to report improvements in HRQoL. Conversely, the longer follow-up of the traditional implant patients in the current study might be expected to display a relative worsening of HRQoL as compared to the shorter MCGRs follow-up, with longer time to experience repeated surgical lengthenings and/or device failure. With no significant differences between groups, the traditional implant group could be considered to have a greater impact on HRQoL than MCGRs, but our study was not sufficiently powered to show this effect.

Disease severity may also have a role to play in why significant differences in HRQoL—with the exception of activities of daily living for all patients—were not identified in the current study. In a study from Germany, although no statistically significant differences in EOSQ-24-G (German version) were noted between VEPTR and MCGRs groups, those with a neuromuscular scoliosis (i.e., CP) and non-ambulatory status (i.e., GMFCS V) showed significantly lower HRQoL scores both at pre-index surgery and the final follow-up [15]. Given that the majority of the children in the current study were GMFCS V, it may be that concomitant factors outside of the spine (e.g., medical co-morbidities) or secondary musculoskeletal problems (e.g., hip displacement, muscle/tendon contractures) have a greater impact on overall HRQoL than neuromuscular scoliosis [1,16,17].

Another potential reason for the lack of HRQoL improvement seen may be related to curve flexibility. The primary indications for performing scoliosis correction for older children with non-ambulatory CP is to improve sitting balance and head control/supports, reduce pain, and prevent decubiti [18]. These issues become more problematic as the curve stiffens with growth. In younger children, the curves are relatively flexible, thus more easily accommodated with adaptive wheelchair seating systems, with or without a soft orthosis, even without corrective surgery [19,20,21]. Hence, growth-friendly implants for EOS in CP may not improve these issues over and above what non-operative solutions can offer at a younger age. In our study, both traditional implants and MCGRs had small positive mean absolute differences for physical function (which includes an item on sitting) and pain/discomfort, but these were not found to be statistically significant (Table 3).

There were several strengths to our study. HRQoL outcomes have not been previously studied in CP patients treated with growth-friendly implants for EOS. All patients included had the same single etiology for their scoliosis, whereas most studies on EOS typically involve patients with a heterogeneous mix of etiologies. This study was comparative (Level III evidence), which is a strength, since most evaluations of EOS are conducted without a control or comparison group (Level IV evidence). The use of a validated HRQoL measure developed for EOS patients, the EOSQ-24, had not been previously utilized for children with CP and EOS. Like the CPCHILD, the EOSQ-24 was designed for caregivers, as children nine years of age and younger, especially those with severe CP (GMFCS IV–V), would have difficulty completing a HRQoL questionnaire. Respiratory function is an issue for children with pediatric scoliosis [22,23]. This was certainly an issue for our cohort of patients with pre-operative and post-operative EOSQ-24 mean scores for the pulmonary domain found to be less than 70/100.

The limitations of our study are that it was multicentered, included a small sample size, some questionnaires had uncompleted items, it used questionnaires, and we utilized the EOSQ-24 instead of the CPCHILD. The CPCHILD is the preferred tool for children with non-ambulatory CP, though not specifically designed for the early-onset age group [9]. Furthermore, different institutions may have varying procedures and methods for collecting data, which may have contributed to the reduced number of patients included in the current study. Due to the retrospective nature of our study, only results from the EOSQ-24 were available for analysis, as it is the tool employed by our multicenter pediatric spine study group as a standard HRQoL measure for EOS patients. However, the EOSQ-24 is a valid, reliable, and responsive tool for evaluating HRQoL in EOS patients [11].

Future work should include a prospective study of similar patients using the CPCHILD tool and aim to obtain a larger sample size. While the results of this study may not have been statistically significant, there is potential for clinically significant findings. Research is currently being conducted to define the minimal clinically important difference (MCID) of the EOSQ-24. Future controlled studies involving patients that underwent no surgery for their EOS would be of benefit to assess the natural history of HRQoL in CP as it relates to neuromuscular scoliosis. A systematic review of severe adolescent idiopathic scoliosis (AIS) patients found that significant improvements in SRS 22r scores following surgery were small and probably clinically insignificant when compared to a non-operative group [24]. Similar research on EOS secondary to neuromuscular disorders (i.e., CP) would be beneficial.

## 5. Conclusions

At the minimum two-year follow-up, CP patients had small, but statistically non-significant, improvements in HRQoL following growth-friendly implants for EOS. Compared to MCGRs, traditional implants provided a modest additional benefit in HRQoL. This data should improve precision and personalized medicine for this patient population.

## Figures and Tables

**Table 1:** Demographic information. Mean values are reported with 95% confidence intervals. Comparisons between means were conducted using independent samples two-tailed *t* tests with equal variances not assumed. Statistical significance was accepted at *p* < 0.05.

	All Patients (n = 24)	Traditional Implants (n = 9)	MCGR (n = 15)
Growth-friendly implant type	9 traditional/15 MCGRs	5 VEPTR/4 TGRs	15 MCGRs
Gender	13 female/11 male	3 female/6 male	10 female/5 male
Prior treatment	5 bracing/19 none	3 bracing/6 none	2 bracing/13 none
GMFCS level (I–V)	21 V, 1 II, 1 I, 1 unknown	9 V	12 V, 1 II, 1 I, 1 unknown
Age at index surgery (years)	7.4 (6.6–8.2)	7.1 (5.8–8.4)	7.7 (6.6–8.7)
Mean EOSQ-24 follow-up (years)	3.5 (3.0–3.9)	4.4 * (3.7–5.2)	2.9 * (2.6–3.2)

* Indicates significance (*p* = 0.003).

**Table 2:** Radiographic information for patients treated with growth-friendly implants. Mean values are reported with 95% confidence intervals. Comparisons between means were conducted using independent samples two-tailed *t* tests with equal variances not assumed. Statistical significance was accepted at * *p* < 0.05. No pre-index surgery or follow-up values were significantly different when compared between traditional implants and MCGRs (* *p* < 0.05). Some follow-up radiographic measurements are post-definitive surgery. Radiographs were not available for all patients at each visit, as indicated by the n values.

	Traditional Implants		MCGR		All		
	Pre-Index	Follow-Up	Pre-Index	Follow-Up	Pre-Index	Follow-Up	*p* (All)
Age (years)	7.0 (5.7–8.3) n = 9	11.6 (9.4–13.8) n = 4	7.6 (6.5–8.6) n = 15	9.1 (7.4–10.9) n = 6	7.4 (6.6–8.1) n = 24	10.1 (8.6–11.6) n = 10	0.007 *
Primary curve (°)	71.8 (59.4–84.1) n = 9	69.8 (37.3–102) n = 4	86.9 (76.9–96.9) n = 15	53.5 (39.1–67.8) n = 6	81.2 (73.1–89.4) n = 24	60.0 (44.6–75.4) n = 10	0.031 *
Maximum kyphosis (°)	51.4 (38.7–64.1) n = 7	45.5 (27.9–63.1) n = 4	52.8 (42.1–63.6) n = 13	45.6 (28.7–62.5) n = 5	52.4 (44.2–60.5) n = 20	45.6 (34.1–57.0) n = 9	0.356
T1-S1 (cm)	25.5 (22.1–29.0) n = 8	34.4 (31.8–37.1) n = 3	27.1 (25.2–28.9) n = 14	31.1 (28.9–33.4) n = 6	26.5 (24.8–28.2) n = 22	32.2 (30.3–34.2) n = 9	<0.001 *
T1-T12 (cm)	16.6 (14.9–18.3) n = 8	21.2 (19.0–23.4) n = 3	17.1 (16.0–18.3) n = 14	19.1 (17.6–20.6) n = 6	17.0 (16.0–17.9) n = 22	19.8 (18.4–21.1) n = 9	0.004 *

**Table 3:** EOSQ-24 mean absolute differences from pre-index surgery to minimum two-year follow-up. Positive values indicate improvement in HRQoL. Means were compared using independent samples two-tailed *t* tests with equal variances not assumed. Several questionnaires were incomplete. (TI = traditional implants).

Domain	Growth-Friendly Implant Type	Mean Absolute Difference (95% CI) from Pre-Index Surgery to Follow-Up	*p* (TI vs. MCGR)
General health	All	+2.7 (−7.1–12.6)	
	TI	+12.5 (−8.7–33.7)	0.236
	MCGR	−2.5 (−12.1–7.1)	
Pain/discomfort	All	+6.0 (−7.2–19.1)	
	TI	+6.3 (−16.0–28.5)	0.977
	MCGR	+5.8 (−11.1–22.7)	
Pulmonary function	All	−0.6 (−17.1–15.8)	
	TI	+7.1 (−27.0–41.3)	0.559
	MCGR	−4.8 (−22.9–13.3)	
Transfer	All	−6.8 (−24.5–10.9)	
	TI	+3.1 (−29.5–35.8)	0.445
	MCGR	−12.5 (−33.5–8.5)	
Physical function	All	+8.3 (−0.9–17.6)	
	TI	+10.6 (−4.1–25.4)	0.694
	MCGR	+6.7 (−5.5–19.0)	
Daily living	All	+23.3 (4.8–41.8)	
	TI	+28.1 (−4.1–60.3)	0.757
	MCGR	+21.6 (−1.6–44.7)	
Fatigue/energy level	All	−1.9 (−15.9–12.2)	
	TI	+8.9 (−16.0–33.8)	0.300
	MCGR	−7.7 (−24.5–9.1)	
Emotion	All	−0.8 (−17.3–15.6)	
	TI	+34.4 (7.2–61.5)	0.030 *
	MCGR	−13.6 (−27.8–0.6)	
Parental impact	All	+7.7 (−2.6–18.1)	
	TI	+15.5 (2.2–28.8)	0.224
	MCGR	+3.0 (−11.3–17.3)	
Financial impact	All	+15.2 (3.4–27.0)	
	TI	+2.8 (−10.0–15.5)	0.070
	MCGR	+23.2 (6.6–39.8)	
Child satisfaction	All	+8.3 (−10.6–27.3)	
	TI	+12.5 (−19.1–44.1)	0.787
	MCGR	+6.8 (−17.1–30.7)	
Parent satisfaction	All	+10.0 (−4.8–24.8)	
	TI	+14.3 (−13.7–42.3)	0.705
	MCGR	+7.7 (−10.2–25.6)	
Total score	All	+4.9 (−3.3–13.1)	
	TI	+9.3 (−4.9–23.5)	0.444
	MCGR	+2.3 (−7.8–12.4)	

* Indicates significance (*p* < 0.05); CI indicates confidence interval.

## Data Availability

Registry data is available to member institutions. Measurements and analysis performed at the IWK Health Centre are on a password-protected server. Access may be arranged through application to the REB.

## Abbreviations

The following abbreviations are used in this manuscript:

HRQoL	Health-related quality of life
CP	Cerebral Palsy
EOS	Early-onset scoliosis
TGR	Traditional growing rods
VEPTR	Vertically expandable prosthetic titanium rib
MCGR	Magnetically controlled growing rod
EOSQ-24	24-item Early-Onset Scoliosis Questionnaire
GMFCS	Gross Motor Function Classification System
PSSG	Pediatric Spine Study Group
